# A Case of Antral Trouble

**Published:** 1900-02-15

**Authors:** J. W. Van Doorn

**Affiliations:** Cleveland, Ohio


### A CASE OF ANTRAL TROUBLE.

BY J. W. VAN DOORN, D.D.S., CLEVELAND, OHIO.

I have a case which is strictly an antral trouble. The patient, a man of fifty-three years or thereabouts, has been for four months on the verge of nervous prostration, incapacitated for work. He has had no trouble with his teeth, therefore had very little occasion to consult a dentist; but after this sickness of his he came to me and complained of pain in the region of the first superior molar. The tooth had been filled, but the filling had dropped out during his sickness. I put on the rubber and worked a little while in the cavity and finally approaching so close to the pulp, found it would be the best thing to destroy the pulp and made an application of arsenic paste for that purpose (Baldock's paste). After leaving in the tooth ten days or two weeks, having had no irritation or pain as a result of application, I removed the paste and entered the pulp chamber without causing any pain. I should have stated that this gentleman had been treated by one of our physicians for nasal catarrh for some time. The discharge from the nostril had been very offensive and great in quantity, and extended over some time. The physician finally suggested that the difficulty might arise from inflammation in the antrum. And so, although I did not know this when the case was presented to me, when I entered the pulp chamber and removed the debris I found that the probe met very little resistance and made its way up at a point about one-half way between palatal and anterior buccal root. I continued the probe and found it extended into the antrum. I enlarged that opening with a large burr and soon succeeded in getting a very copious discharge of muco-purulent pus. I used a three-percent solution of pyrozone, forcing it in until it came out at the left nostril. After thoroughly washing it out in that way for two or three days in succession, I then used glyco-thymoline as a wash. I succeeded by this means in getting rid of the discharge entirely, and it then only became a question of simply closing up the opening. This was done by the use of europhen, pumping it up into the canal and vaporizing it by bringing it into contact with heated instruments, then packing root canals of the tooth and also the artificial canal with the same. The tooth has been sealed up in this manner for about six weeks, and I have seen or telephoned the patient several times and there has been no recurrence of pain or of discharge. Of course it is not cured, but still the antiseptic conditions are thoroughly satisfactory and all things indicate that the inflammation has subsided, and I see no reason why I may not to expect a permanent cure.

